# How to make deposition of images a reality

**DOI:** 10.1107/S1399004714005185

**Published:** 2014-09-30

**Authors:** J. Mitchell Guss, Brian McMahon

**Affiliations:** a School of Molecular Bioscience, The University of Sydney, Sydney, NSW 2006, Australia; b International Union of Crystallography, 5 Abbey Square, Chester CH1 2HU, England

**Keywords:** data deposition

## Abstract

The IUCr Diffraction Data Deposition Working Group is investigating the rationale and policies for routine deposition of diffraction images (and other primary experimental data sets). An information-management framework is described that should inform policy directions, and some of the technical and other issues that need to be addressed in an effort to achieve such a goal are analysed. In the near future, routine data deposition could be encouraged at one of the growing number of institutional repositories that accept data sets or at a generic data-publishing web repository service. To realise all of the potential benefits of depositing diffraction data, specialized archives would be preferable. Funding such an initiative will be challenging.

## Abstract 2

An analysis is performed of the technical and financial challenges to be overcome if deposition of primary experimental data is to become routine.

## 1. Introduction   

Other articles in this series (Kroon-Batenburg & Helliwell, 2014 ▶; Terwilliger & Bricogne, 2014 ▶; Meyer *et al.*, 2014 ▶) have argued the case for the routine deposition of diffraction images and have discussed some of the practicalities and hardware and software requirements for doing so. In this article, we have been charged with discussing how such a goal might be achieved. We note that diffraction data have not always come in the form of an ‘image’, and most of the arguments in this article will apply equally well to other forms of experimental data. We do, however, also touch upon some of the issues that are specific to diffraction images.

The potential benefits of a raw data archive include its utility as a backup facility, in case the principal investigator loses the original data sets; the ability to retry failed structure solutions in the future as processing techniques improve; the possibility of extracting new scientific results in the future from signal in the data not used in a contemporary structure solution; the supply of test data sets to evaluate and refine new software; and safeguarding against gross error or fraudulent practice.

All are valuable to a greater or lesser extent, but the costs of long-term data storage and management are significant, and a proper cost–benefit analysis is desirable. It is clear that there is a greater imperative for archiving data from materials that are difficult or costly to synthesize, or that are destroyed in the radiation beam or are chemically unstable, than is the case for substances that are easy to synthesize and crystallize. On the other hand, the real costs in establishing universally agreed criteria that allow partitioning of raw data into sets that should be retained for the long term and those that can be safely discarded may actually be higher than the hardware costs in simply scaling up storage capacity to retain everything indiscriminately.

This may be particularly difficult to establish in new areas of research using instruments that generate very high volumes of data. The current excitement around the new types of experiments that have been opened up by X-ray free-electron lasers (X-FELS) is a case in point. This is by no means a problem unique to crystallography. The enormous volumes of data generated by particle physics experiments, and latterly by advanced astronomical instruments such as the Square Kilometre Array, demonstrate both that provision (including budget) must be made in the design of new instruments not only for data acquisition but also for data handling, and that compromises may need to be made in the deployment of the instrument to cope at all with the data deluge that follows its commissioning.

With these preparatory remarks, we move on now to outline the considerations that should be brought to bear in attempting to design workable strategies for more-or-less routine deposition of diffraction images.

We begin by describing an ideal goal: some entity that could, in principle, provide all of the requirements discussed by advocates of image deposition. In the real world, such an ideal entity may not be achievable, nor indeed desirable. However, we use this as a starting point in an effort to ensure that the proposals that we develop do not actually work against such an ideal.

This ideal entity, then, which we shall call the Image Archive, is capable of storing all deposited diffraction images with the following attributes.

(i) Diffraction images are available for the long term.
(ii) All images collected for a particular study have persistent identifiers.
(iii) The persistent identifiers allow the status of the images to be ascertained indefinitely (*i.e.* if deposited images are subsequently deleted or corrupted, attempts to locate them by use of the persistent identifier should return information about what has happened to them).
(iv) Images are accessible through their persistent identifiers.
(v) Where images are not publicly available because of embargo or intellectual property rights restrictions, attempts to access them through their persistent identifiers should return information about the restrictions on their distribution or use.
(vi) Publications and other scientific output resulting from the use of images should be linked to them; ideally, such linking should be bidirectional.
(vii) Images should be discoverable through searches against a wide variety of properties, *e.g.* chemical composition of the sample; crystallographic properties (unit-cell dimensions, cell system, space group *etc.*); dates and times of the experiment; facilities/beamlines; experimentalists; beam characteristics; experiment types.
(viii) Verification should have taken place to check that the deposited images are indeed what the depositor claims. At present, this is a difficult thing to guarantee. In principle, the archive curators could reprocess and perhaps even reduce the data to calculate an *R*_merge_ with an author’s *F* or *I* values, but this would, of course, involve substantial computing resources. Such a burden could be reduced by persuading software writers to facilitate such verification through the appropriate use of metadata written routinely as part of any output file. (These metadata might be ‘scientific’ in nature, or might be purely computational, such as checksums that verify the identity of individual data sets.)

## 2. The archive   

In §[Sec sec1]1, we characterized our ideal data-deposition facility as an archive. By this we mean specifically a preservation framework conforming to the Reference Model of the Open Archival Information System (OAIS; Consultative Committee for Space Data Systems, 2002 ▶) developed initially for the space-science community.

An early draft of this reference model was used in drawing up the IUCr policy on archiving its journal publications (http://journals.iucr.org/services/archivingpolicy.html). The latest revision of the Reference Model (Consultative Committee for Space Data Systems, 2012 ▶) has been upgraded from a Recommended Standard to a Recommended Practice, demonstrating its practical benefits to the space-science community, which has amassed considerable experience in storing and managing large volumes of data. Although this is a community with a strong history of collaboration at the data level, we believe that the OAIS reference model can be used equally effectively to construct an archive where access to some content may be restricted or embargoed.

Among the characteristics of open-archival information systems that are particularly relevant to the requirements of the crystallographic community are the following.

### 2.1. Designated Community   

From the outset, the OAIS documentation emphasizes that an open archival information system serves the needs of a ‘Designated Community’; that is, a particular implementation can (and should) be tailored to the requirements, discipline knowledge, tools and common practice of a specific community. It is understood that an OAIS is, at some level, managed by an organization that has accepted the responsibility to preserve information and make it available to the Designated Community.

The OAIS Reference Model defines the Designated Community as ‘an identified group of potential Customers who should be able to understand a particular set of information’, and notes that this may include multiple user communities. It is interesting that in the Reference Model, the ‘Designated Community is defined by the Archive’, a definition that ‘may change over time’. This alerts us to the need, when attempting to design an archive for diffraction images, to consider future applications extending to communities other than those we currently think of as using or needing such data. We need to specify and capture metadata that will assist discoverability by other communities, and we will need in due course to extend the metadata specifications to accommodate the additional requirements of those communities. Consequently, the archive must retain a capacity for extensibility, in function as well as in sheer volume.

Parenthetically, the Designated Community in our case will include the data depositors (or their laboratories) who wish to use the archive as a means of preserving their own data for their own use. Such users may be adequately served by a model which retains a minimal set of descriptive metadata for deposited data sets, since it can be assumed that the depositors will retain (for at least some time) the additional knowledge needed to reprocess or otherwise use their own data. Such users could be served by a more lightweight preservation strategy than we are trying to encourage. However, such an approach is likely to be workable only over the short term. A depositor wishing to return to experimental data collected years previously may, unless unusually assiduous in record-keeping, be heavily dependent on the metadata associated with the old data sets at the time of their deposition.

A vivid account of the practical difficulties in reanalyzing image data without complete knowledge of the instrumental metadata is provided by Kroon-Batenburg & Helliwell (2014 ▶).

### 2.2. Long-term value   

In the OAIS Reference Model, it is assumed that the information being maintained ‘has been deemed to need Long-Term Preservation, even if the OAIS itself is not permanent’ (Consultative Committee for Space Data Systems, 2012 ▶). The definition of long-term covers time spans that are ‘long enough to be concerned with the impacts of changing technologies, including support for new media and data formats, or with a changing user community.’

Hence, ‘long-term’ does not necessarily imply ‘forever’ (although it may do, depending on the value that the community assigns to the information). Indeed, it does not even necessarily imply a time span extending over very many years. If information is captured in proprietary formats that change frequently because of rapidly evolving technologies, then the time between capturing raw data and publishing a considered account of the research results from that data may already constitute ‘the long term’ so far as preservation requirements are concerned.

### 2.3. Access and use   

For maximum benefit to a wider community, it is important that deposited data are accompanied by sufficient metadata to allow discovery, interpretation and re-use. We discuss some of the technical requirements for such metadata in a later section. However, we note here that for correct ‘interpretation and re-use’ there will need to be requirements for ‘minimum sets’ of metadata characterizing the experiments, data formats and processing techniques. For derived structural models, the crystallographic community already prescribes such minimum sets, for example in journal *Notes for Authors* (IUCr Editorial Office, 2012 ▶; Einspahr & Weiss, 2008 ▶), in methods-specific Validation Task Forces convened by the Worldwide Protein Data Bank for macromolecular structures (Read *et al.*, 2011 ▶; Trewhella *et al.*, 2013 ▶; Montelione *et al.*, 2013 ▶; Henderson *et al.*, 2012 ▶) or as recommendations of IUCr Commissions (Chapuis *et al.*, 1997 ▶). Some IUCr Commissions are now beginning to address the metadata requirements for experimental data sets (Ravel *et al.*, 2012 ▶; Jacques *et al.*, 2012 ▶).

There have also been informal community-driven efforts to define a minimal set of metadata required to process diffraction images from different detectors writing CBF/imgCIF files (Bernstein & Hammersley, 2005 ▶): the so-called ‘miniCBF’ set developed initially for PILATUS detectors (Bernstein, 2007 ▶).

A delicate question is the restriction of access to individual data sets. There is a general sense that research data, certainly where generated from publicly funded research, should eventually be ‘openly available with as few restrictions as possible’ (RCUK Common Principles on Data Policy; http://www.rcuk.ac.uk/research/Pages/DataPolicy.aspx). Neverthe­less, there are well established embargo practices that allow researchers privileged access to their own data for some time. In order to manage authorized re-use of deposited data sets, an archive must be able to handle varying embargo periods (perhaps set by different funding-body mandates). This will, naturally, involve recognition of individuals who do have access rights (*i.e.* the depositor). Such recognition could be facilitated by use of one or more of the emerging protocols for researcher identification, such as ORCID (http://orcid.org).

Many data-embargo practices implicitly assume that the deposited data will lead to a research publication and the eventual disposition of the data is linked to the time and the manner of publication. However, archives established primarily for the early deposition of experimental data will have to cope with the accumulation of data sets that do not become associated with a publication. Policies need to be put in place to ensure that such ‘orphaned’ data sets may eventually be re-used. A precedent for this exists in the UK National Crystallography Service (Coles & Gale, 2012 ▶), where users are encouraged and assisted in the public release of their data after three years if no publication has resulted. A precedent also exists in the PDB, where there are now many depositions with no associated publication and all depositions have a finite hold time, whether published or not.

## 3. Centralized and distributed deposition   

In considering how the objectives for an ‘ideal’ archive might be realised in practice, we discuss the relative advantages and disadvantages of centralized *versus* distributed solutions. In this context, ‘centralized’ really means that an archive facility is managed either by a single body or organization or by multiple partners operating under a single set of rules and protocols. In this view, the Protein Data Bank (PDB) would be considered as a ‘centralized’ archive. Although it consists of database centres in three different continents, the three sites host actual mirrors of the underlying database, albeit with different portals for access. The partners collaborate on improving the quality, integrity and consistency of the contents of the PDB archive. They do, however, act as ‘friendly’ competitors in terms of ‘data-out’, and they use different database technologies and provide their own sets of unique services (Berman *et al.*, 2003 ▶; Gutmanas *et al.*, 2013 ▶).

### 3.1. The advantages of a centralized facility   

The immediate advantage of a centralized facility (in the sense just mentioned) – that is, a single point of deposition for all diffraction images worldwide – is that it can most easily exert central control over its holdings. It can mandate deposition formats or apply uniform translation or preservation approaches to particular types of data. In particular, if it is focused on the requirements of one specific discipline, it is optimally attuned to the requirements, current and emerging practices of a specific ‘Designated Community’.

It can also provide efficient search and data-mining facilities across a complete corpus of data sets. In principle, such services can be run over a network of distributed nodes. In this case, however, the efficiency and completeness of such a service would depend on the degree of similarity of the data-storage schemas in use at the different nodes, or on the establishment of another consolidated metadata database to bridge the different originating schemas.

A centralized archive can benefit from certain economies of scale. These can be hardware-related: a single facility serving all of crystallography could calculate its data-storage requirements based on the volume of publications, numbers of structural results deposited in existing structural databases and close liaison with experimental facilities and detector manufacturers.

It can also benefit from a concentration of expertise within a staff complement specializing in the discipline’s requirements and needs.

### 3.2. The economic challenge of a centralized facility   

The most obvious disadvantage for a facility specializing in the deposition of diffraction images (or other large experimental data sets) is the need to raise a new funding stream to cover the additional hardware, software development and personnel requirements. It is worth remarking that the total costs for developing a dedicated central facility might very well be significantly lower than the costs worldwide of achieving the same result through distributed effort. For example, cataloguing or synchronizing collections at many diverse locations may require new protocols that would simply not be needed if all of the data were managed within a single storage facility. Nevertheless, the need to secure a significant level of funding for a single facility poses an economic challenge.

To explore this economic challenge further, consider some possible models for a central archive. Remember that in each case the archive might itself be spread over several geographical locations and involve different participating bodies, but it will work within a common operational framework. It is also likely that the participating bodies, if there be more than one, share a common, or at least broadly similar, funding framework. For example, it would be difficult if some nodes were publicly funded and others applied a ‘user-pays’ model, especially if all data sets were open to free access.

Whenever we refer below to existing crystallographic databases, we do so to identify concrete examples with which the reader may have some familiarity and speculate on approaches that these bodies might take. At this point in the public debate, we are not recommending any particular course of action or ruling out others that we do not mention at all.

#### 3.2.1. Extension of an existing publicly funded crystallo­graphic facility   

An example of a publicly funded archive of data specific to one experimental technique is the Biological Magnetic Resonance Data Bank (BMRB) at the University of Wisconsin-Madison, which acts as a depository for NMR data from peptides, proteins and nucleic acids. It takes seriously the characterization of data using the NMR-STAR standard format (Ulrich *et al.*, 1989 ▶) and is active in defining and improving data-quality metrics (Montelione *et al.*, 2013 ▶).

There is no corresponding archive of crystallographic experimental data, and it has been suggested that it would be interesting to explore the option of creating a new organization dedicated to storing X-ray data, analogous to BMRB (Gerard Kleywegt, personal communication). We explore some of the relevant issues in §[Sec sec3.2.4]3.2.4. Given the current funding pressures on the BMRB (its current grant from the National Library of Medicine will not be renewed after late 2014), we believe that very intensive lobbying would need to be brought to bear on public funding agencies to bring this about.

It might be easier to make a case for broadening the existing remit of the PDB, which, although agnostic to the particular techniques leading to macromolecular structural models, does hold a very high proportion of crystallographic results, and does archive associated structure factors. Any such case would, however, need to be carefully articulated and demonstrated to be a legitimate and worthwhile extension of the PDB’s current mission.

Would all data sets collected be retained forever, or would there be strategies for disposing of data sets that did not in time lead to a structure publication? Data-retention policies will have a significant impact on the actual hardware and maintenance costs. For example, Westbrook (2012 ▶) demonstrated that handling 50 TB of raw data per year (equivalent to around 10 000 data sets of average size 5 GB) with the PDB’s existing standards of archive security could involve annual costs in excess of $1 million, depending on the storage medium used. This assumes the current PDB practice of storing multiple copies at each archive site. For image data, it might be considered sufficient to store this as multiple copies at just one PDB site or as single copies at two or three sites.

Using commercial cloud-storage facilities would reduce this cost substantially. However, retaining the same level of confidence in the long-term survivability of an archive might involve duplicating data with multiple commercial vendors, and the costs begin to rise again. If one could identify data whose retention was desirable, but whose accidental loss could be considered acceptable, then one would need less hardware redundancy, and again costs could be lowered.

Hence, lobbying for public funding would involve the identification of the policies that the crystallographic community thought most appropriate and a detailed cost–benefit analysis to persuade public bodies that the required level of investment was in the public good.

#### 3.2.2. Extension of an existing user-funded crystallographic facility   

Here, an exemplar might be the Cambridge Crystallographic Data Centre. In this case, the retention of experimental data could be seen as an extension of the CCDC’s activities. It is likely that the necessary large upscaling in hardware costs to retain diffraction images would significantly increase the overall production costs for the Cambridge Structural Database (CSD). The question would then arise of how easily the CCDC could pass on such costs to its users in the contributions that it needs to maintain the CSD.

It may be that the raw data sets could be monetized separately as a new product, and charged for on a straightforward cost-recovery basis. This would certainly test the market value of such data sets. It would also require a detailed commercial assessment of the likely market. We are not in a position to pre-judge the likely result of such an analysis. However, if the most successful business model involved the sale of small numbers of data sets at high cost (as would be necessary to cover the full costs of retaining and managing large volumes of data), this might give rise to a number of concerns. Would this not severely restrict access to these data sets? This would seem to run counter to current trends towards making raw data openly available (Royal Society, 2012 ▶).

It might be possible to avoid such concerns by seeking some public subsidy of this part of the CCDC’s work. This, however, would involve new directions in the management of such an operation, and a need to persuade public funding agencies, who do not directly fund the archiving of even the structures themselves, to collaborate in such a way.

#### 3.2.3. Use of a domain-independent web service   

As national research data-management policies become established, so we are seeing a growth in the number of services which offer file storage and archiving services suitable for research data sets, *e.g.* Dryad (http://www.datadryad.org), ResearchGate (http://www.researchgate.net) and figshare (http://www.figshare.com).

There are certain obvious attractions to using such a service: an external organization has responsibility for maintenance of the storage and preservation infrastructure, and the service providers have a strong incentive to capture and categorize basic identification and other characterization metadata. Since the services are domain-independent, they are unlikely to provide sophisticated metadata services that would allow targeted searching or analysis of deposited data sets. However, it is possible that value-added services tailored for specific disciplines could be provided by third parties, so long as the data sets of particular interest to those third parties (in our case, diffraction images or other crystallographic experimental data) could be cleanly identified. We suggest some ways in which this could be achieved in §[Sec sec4]4.

However, most such initiatives currently assume that depositors will have either small data sets (in terms of file size) or larger ones that are deposited infrequently. The data-publishing charges announced by Dryad in September 2013 (of the order of $70 per ‘data package’) would be significant for high-volume crystallographic research. In the terminology of Dryad, a data package comprises the entire set of data files associated with a single publication plus the metadata describing the combined set. For data packages larger than 10 GB, an additional charge (of the order of $10 per gigabyte) is payable.

It is still rather early to assess how these initiatives will fare in the longer term. If one provider were to become predominant, or were seen to offer cost-effective services particularly well suited to our requirements, it would be useful for the crystallographic community to work with that provider and explore ways of optimizing its functionality (and minimizing the cost burden). If several providers were to enter the market, the benefits of a single centralized service would be lost, unless all crystallographic depositors could be persuaded to use a single provider.

However, since such services do now exist, it is possible that some crystallographic data sets will begin to be deposited with them. In that case, they should be considered as components of an initially informal distributed resource, which may also contain institutional repositories (IRs) or other *ad hoc* solutions.

#### 3.2.4. Creation of a new user-funded crystallographic facility   

Our discussion in §[Sec sec3.2.2]3.2.2 of extending the operating model of an organization such as CCDC touched upon the difficulties of charging for the supply of raw data sets within a commercial framework. However, the existence of service providers as outlined in §[Sec sec3.2.3]3.2.3 suggests that there might be a role for a discipline-specific facility to provide archiving, curation and data-management facilities as a service to the community as a whole. The intention would be to make the deposited data sets freely accessible (once any relevant embargo period had passed), so that there would be no income from ‘sales’ of data sets *per se*.

It may also be the case that such a new initiative could embrace all areas of crystallographic research: macromolecular, small-molecule, inorganic, single-crystal and powder, X-ray, neutron and electron diffraction. The advantages would include the creation of a single resource across all of crystallography and the potential to generate revenue from the widest spectrum of ‘clients’ or ‘customers’. Disadvantages could include the need for a significant number of staff to provide expertise across many fields, the possibility of greater growth than planned (*e.g.* into other fields such as NMR or tomographic imaging that move progressively further from the original crystallographic emphasis) and the possibility of competition with other generic data-deposition services such as Dryad.

These latter factors, of course, might be seen simply as the market forces to which any commercial enterprise needs to adapt. While establishing the challenges for a successful operating model, they do not in themselves rule out the possibility of launching and developing such a facility.

We anticipate that a significant source of income for such a facility could be from organizations such as synchrotrons, neutron sources or other large laboratory facilities, databases, journals and possibly university laboratories, who would themselves be relieved of direct costs.

An alternative (or possibly complementary) scheme would levy charges directly on an author/depositor, by analogy with the ‘Gold’ model of open-access publication. An interesting question (in the current science economy where career advancement and reward are linked heavily to a researcher’s publication record) is whether such an upfront payment would have a negative effect on the deposition of data unrelated to a publication.

These concerns are not unique to crystallography, and we follow with interest the development of organizations such as DataCite and Dryad which are trying to establish a working model for ‘data publication’ to be regarded in itself as a driver for academic recognition and scientific career development. The challenge we offer to our more entrepreneurial colleagues is to explore the feasibility of creating and running a central repository/archive for all crystallographic experimental data as a sound business proposition.

### 3.3. Other disadvantages of a centralized facility   

A general disadvantage of any data-deposition service outside of an experimental facility is the network transfer overhead. Although many facilities hosted by, or sharing, academic facilities would be able to benefit from high-speed research networks, other contributing sites might have access to lower bandwidth networks. In any case, the bottleneck for network transfer will always be the bandwidth of the slowest segment traversed, and this may always be a problem for sites connecting to a single geographic location.

Network transfer problems can be ameliorated by having a multi-site instance of a central facility (as with the PDB) or by using distributed cloud storage, possibly from one or more commercial vendors with global reach.

One supposed advantage of a central facility – that it provides a centre for domain expertise – may turn into the disadvantage of overspecialization, especially if other domains build parallel archival systems or if currently generic solutions such as Dryad grow to accommodate the specific needs of many different domains, but in a way that excludes or fails to interoperate effectively with crystallography. The best guard against this is to ensure that any central facility retains an active interest in, and liaises with, similar initiatives outside of the crystallographic community.

### 3.4. The advantages of a fully distributed approach   

Given the challenges of the centralized approach – especially the financial challenge, but also the current lack of a specific set of policies – we should consider the implications of beginning to deposit raw data sets using the existing and near-future scenario of repositories of different scale and purpose.

The first advantage is, of course, that of low start-up cost. In many cases there may already be existing informal arrangements for data storage, *e.g.* at synchrotron or neutron facilities or in university repositories, that make adequate provision for data retention at least over the timescale of a grant funding cycle. Although these may provide little long-term security, emphasizing their short-term importance may help to raise awareness of the importance of secure and orderly data retention in the minds of researchers. If researchers become used to the routine deposition of metadata that will allow them to retrieve and re-process their own data on this timescale, they may become accustomed to the more stringent requirements for metadata capture needed for longer term preservation.

This approach can also build on the growing number of research data-management policies being established under pressure from government and other funding agencies.

Such ‘local’ solutions may also use protocols which are already familiar to the researcher. For example, if a researcher is used to depositing publications in a university repository, and that repository extends its holdings to include data sets, no new skills may need to be learned.

### 3.5. Disadvantages of a fully distributed approach   

On the other hand, the proliferation of local protocols is likely to be associated with a low level of standardization across all of the possible contributing repositories: IRs, synchrotron and neutron facilities, Dryad, figshare *etc.*

There is also likely to be great variability in the retention policies of each separate institution. This will affect not only the length of time for which data sets are retained, but also the types of data and the reason one might want to retain them. In crystallography, for example, one might in the long term wish to differentiate between the deposition of data sets associated with publications and those that are collected during preliminary or incomplete studies. On the other hand, one might wish to retain data sets associated with unsolved structures in the hope that future solution may be possible with new software.

It will also be more difficult to locate or discover data sets that are spread across a wide range of repositories. This can be ameliorated by promoting common metadata standards (as discussed in §[Sec sec4]4 below), but it may also be necessary to establish third-party aggregators or secondary databases to provide common discovery and access mechanisms. If this is not performed, large volumes of data may be locked up at many different locations, forever unvisited in spite of their potential value.

Finally, across a large number of repositories there will inevitably be a wide variability in the expertise of the repository managers and archivists.

## 4. Promoting interoperability   

Although there are a large number of disadvantages to the ‘fully distributed’ approach, it is likely on financial grounds that it will predominate at least in the short term. One should therefore identify the most important aspects that need to be addressed to smooth the transition to an effective OAIS solution in the longer term.

We comment on the absolute need for a standard system of persistent identifiers to locate data sets stored in many different places and possibly under many different database-management systems (§[Sec sec4.1]4.1).

We also discuss metadata requirements. ‘Metadata’ is a common buzzword in any discussion of information management, but is a rather fluid term. We like the notion that ‘metadata is the data that *you* are not interested in’. Certainly, there is no formal distinction in CIF dictionaries between data items that describe experimental observations (for example, intensities) *versus*, let us say, author names and affiliations or the chemical composition of a sample. This allows all relevant information to be stored in the same file, which itself can be a great help in the practical aspects of archiving.

In practice, we should probably be concerned with at least three categories of metadata. The first, which we call ‘characterization’ metadata, refers to rather general characteristics of any data set and might be expected to be captured by the ingest process of any discipline-agnostic data repository; for example, depositor names and affiliation, grant numbers, purpose of study. These form the subject of §[Sec sec4.2]4.2.

The next category contains the technical information required to interpret the images, including the format, instrument type and parameters. We will call these ‘interpretation’ metadata, and refer to them in §[Sec sec4.3]4.3. Their purpose is to provide sufficient information to allow at least an understanding of the nature of the experimental data in an image or other data file. For an image downloaded by the original depositor, who still has access to the original laboratory notebook and files, this may be sufficient to allow subsequent re-processing and reanalysis based on the original experiment.

For other users, however, this will not be sufficient. To allow third parties to reanalyse the stored data effectively, further information needs to be captured and stored, including a complete description of the sample and how it was obtained. It is metadata in this category that should be prescribed in the ‘minimum set’ initiatives discussed in §[Sec sec2.3]2.3. We might call this category ‘metadata for reproducibility’.

### 4.1. Discoverability through persistent identifiers   

A common system of unique and persistent identifiers ensures the long-term discoverability of individual data sets referenced through these identifiers.

We suggest that there are benefits in the general community adoption of one specific persistent identifier scheme: the digital object identifier (DOI), an international standard (International Organization for Standardization, 2012 ▶) managed by the International DOI Federation. This is supported by an extensive international infrastructure and can be used alongside other identification systems if required. It allows great flexibility in the form of the identification string used, so that any existing well defined scheme of identifying data sets locally can be used as the basis for a DOI.

A DOI may be registered through one of a number of registration agencies (RAs). Historically, journal publishers have used CrossRef (http://www.crossref.org) to register DOIs for articles, while DataCite (http://www.datacite.org), a younger organization, specializes in handling scientific data sets. However, there is close cooperation between these two RAs, and many crystallographic data sets (such as structures in the PDB) have had DOIs registered through CrossRef.

One advantage of a well established infrastructure is that procedures are already in place, for example for transferring ownership of DOIs between ‘publishers’ (in the established terminology, anyone entitled to register a DOI is known as a publisher). This may be useful in a model where scientists initially deposit all their data sets with a local institutional repository (IR) (where the institution would be the ‘publisher’), but data sets associated with a subsequent publication might be transferred physically to a new location for long-term archiving. In this case, the ownership of the DOIs could be transferred to the new archive.

It would not be necessary to transfer ownership of DOIs in a slightly different model, in which a central facility aggregates the metadata associated with published data sets but the data sets themselves remain physically on the IR. In such cases the ownership of the DOI should remain with the organization that has most control over the location of the data, since it will be best placed to update the location information if the data set is moved to a new server or associated with a new URL.

A disadvantage of a DOI is that the registration process costs money: not a large amount per data set (typically a few cents up to a dollar), but an appreciable cost if very large numbers of data sets are deposited. The costs may be ameliorated depending on what is considered as constituting a ‘data set’. In the crystallographic context, should one assign one DOI per frame? per scan? per session? per experiment? per structure? Each level of granularity has its advantages, in terms of precision, and its disadvantages, in terms of registration cost and management complexity. It is likely that the community will converge on a common practice that minimizes the number of DOIs issued; but it will then need to consider the problem of identifying particular subsets of the data set that was registered.

There are already precedents that will undoubtedly guide early practice and recommendations (for example, the data-management practices and DOI registration procedures at Diamond Light Source or the Australian Synchrotron). On the other hand, novel techniques or types of experiment might demand different strategies (for example, as applications of X-ray free-electron lasers become more diverse).

The problem of granularity of data citations is discussed in a recent report (CODATA–ICSTI Task Group on Data Citation Standards and Practices, 2013 ▶). A possible approach to ‘deep citation’ (the referencing of subsets of an information object identified by a DOI) is the use of OpenURL (Van de Sompel & Beit-Arie, 2001 ▶) to convey a query payload to the resource identified by the DOI. An implementation of this approach by CrossRef allows deep citing of content within *International Tables for Crystallography* Online (http://it.iucr.org) and may be a useful model for further investigation in the context of data sets.

The role of the DOI is to ensure persistent ‘discoverability’ of a data set (or, indeed, any other object). This may, legitimately, mean a statement that the actual data have been deleted: at least the person trying to access the data knows what has happened to them. Assigning a persistent identifier does not mandate or guarantee that the object referenced will be accessible forever.

### 4.2. Discoverability through common metadata   

While we might expect that many data sets will be accessed from links out of published articles, one can envisage many circumstances where it is interesting to search for unknown data sets. In order to provide effective search engines, it will be necessary to develop standards for ‘characterization’ metadata. These will allow an end user to locate a data set through some common criteria supported by a diverse range of distributed repositories, *i.e.* name of creator, creation date *etc*.

Most repository platforms support a metadata standard known as Dublin Core (http://dublincore.org) that can be considered to be the lowest common denominator. However, Dublin Core (DC) has limited use in creating search engines that would work on specific items of scientific data. Distributed repositories must support DC as a common base, but there is also a need to build richer metadata schemas characterizing scientific data sets at a more granular level. Inter-repository protocols such as the Open Archives Initiative Protocol for Metadata Harvesting (OAI-PMH) exist (http://www.openarchives.org/pmh/) that allow the use of alternative metadata schemas through negotiation.

We would therefore recommend that depositors try to ensure that they use institutional repositories (or other deposition platforms) that support OAI-PMH. The name ‘Protocol for Metadata Harvesting’ suggests that such repositories may be harvested by third parties who are interested in providing aggregation and possibly search services. Many generic services of this type do exist (*e.g.* OpenDOAR; http://www.opendoar.org). However, it should be possible to build at least a directory of repositories containing crystallographic data sets, and perhaps a search engine, by using some of the features that OAI-PMH supports but which are as yet little used.

The first such feature is the characterization of different types of content within a repository using the optional ‘set’ construct. Because this permits arbitrary classification of the components of a set, the crystallographic community should draw up an approved terminology which would allow OAI-PMH repositories to be selectively harvested for specific types of data set, such as ‘crystal structure model’, ‘X-ray diffraction image’, ‘powder diffraction profile’ *etc*.

The second is the negotiation feature that allows repositories to expose metadata other than Dublin Core. To be effective, again the community would need to establish common sets of metadata that would yield the necessary information for identifying and harvesting data sets of crystallographic interest. Because there are many ways of loading material into IRs (and given that many such repositories already in existence have little flexibility in the way that they obtain information about the uploaded content at the time of ingest), it is likely that such a metadata set will have to be quite small. Of course, if the data sets themselves are richly annotated, *e.g.* because they are in CIF, CBF or NeXus formats richly populated by laboratory workflow systems, software packages or author annotation, then the harvester can build a much richer database for use by the crystallographic community.

An existing example of the design of repository-friendly metadata is the preservation metadata specification for the eCrystals platform (Patel, 2009 ▶).

### 4.3. Ensuring long-term decipherability   

The more disparate repositories that exist, the more chance there is that proprietary binary formats become detached from scientifically meaningful metadata and end up as indecipherable (but often very large) blobs filling up hard drives. Crystallography is fortunate in having very well documented archival formats (CIF dictionaries; Hall & McMahon, 2005 ▶). For image data, CBF exists as a fully documented binary working format; imgCIF, a fully ASCII encoding, is even more robust (Bernstein & Hammersley, 2005 ▶). The best way to ensure long-term storage and re-use would be for the community (working with instrument manufacturers) to create robust conversion tools from their proprietary formats to imgCIF or, better, to adopt native output to CBF or imgCIF.

However, there are speed/performance issues, especially with latest-generation large detectors, that will see other formats used in the near future. The existing practice at beamlines at Diamond Light Source (DLS) is to write images from PILATUS detectors in CBF format. This has been a successful strategy as DLS has led the way in pushing the limits of format standardization and data retention with the highest-performance detectors currently available. Neverthe­less, the next-generation DECTRIS Eiger is capable of writing compressed data at a rate of 18 Gb s^−1^ (Table 1 ▶) and is expected to write output using the HDF5 format that can provide the virtual file system necessary to manage the massive data flow (Bernstein *et al.*, 2013 ▶).

This is not necessarily an insuperable difficulty if the output HDF5 files conform to a suitable NeXus application definition (see the following section), because this will result in another well defined and documented format. However, the imgCIF dictionary is well tuned to the mmCIF dictionary, and for purely crystallo­graphic applications there may be benefits to having the raw and derived data sets in the same formalism. It is possible, therefore, that there may be some future benefit in translating images captured as NeXus files into imgCIF archive files for some subset of data that are considered important to preserve for a long period of time.

Kroon-Batenburg & Helliwell (2014 ▶) give a good account of the metadata that should be stored alongside a diffraction image to permit its interpretation by contemporary processing software. This is the type of metadata that we called ‘interpretation metadata’ in our introduction to this section.

### 4.4. Format issues   

Interoperability at the level of ‘Dublin Core’ metadata may help with the storage and retrieval of specifically identified data sets from distributed IRs. However, ‘added-value’ interoperability outside the crystallographic community (federated search portals, extraction of subsets of large data sets, establishment of automated procedures for expiring data sets, linking to publications, sorting by different criteria *etc.*) may require more.

It may be useful to establish appropriate XML or RDF representations of CIF data to facilitate integration with repository systems built on semantic web technologies. There are already XML schemas for CIF, *e.g.* the PDBML schema for data exchange between the members of the wwPDB consortium (http://pdbml.pdb.org) and an XML schema in use in the Bilbao Crystallographic Server (http://www.cryst.ehu.es/cryst/xml). However, these are tailored to very specific purposes and it is likely that other schemas would need to be designed to integrate with applications from other scientific disciplines.

In the context of experimental facilities, concordances may need to be set up with other standard data formats used within the facilities. For example, if beamline images are captured as HDF5 files (as discussed in the preceding section), it would be beneficial to have a high level of compatibility between the resulting files (using a NeXus content model) and imgCIF or CBF files. At a satellite workshop of the 28th European Crystallographic Meeting in August 2013, the committees responsible for managing the CIF and NeXus standards (COMCIFS and NIAC, respectively) agreed to develop a draft concordance between the two formats and to work towards a complete mapping for macromolecular synchrotron beamline applications (Bernstein *et al.*, 2013 ▶; https://www.sites.google.com/site/nexuscbf/mapping-draft).

## 5. Case studies   

We rapidly survey some existing initiatives that can provide useful experience and pointers for future development.

### 5.1. TARDIS   

TARDIS (http://www.tardis.edu.au) is an existing network of federated repositories at a national (Australian) level (Androulakis *et al.*, 2008 ▶). Its original scope is expressed in its name, an acronym for ‘The Australian Repositories for Diffraction ImageS’. Its characteristics of particular interest here may be summarized as follows.

The initial purpose of TARDIS was the storage and dissemination of public data sets containing diffraction images. In practice this involved acting as a metadata aggregator, with actual data storage distributed at the depositor’s local laboratory or institution. Data sets were registered on the TARDIS site for purposes of search, download and citation. It used a technical metadata schema based on the Scientific Metadata Model of CCLRC (now the UK Science and Technology Facilities Council; Sufi & Mathews, 2004 ▶). Multiple files comprising a complete data set (together with other relevant ancillary files) were packaged into a composite information object described by the Library of Congress METS schema (Cantara, 2005 ▶). This fulfils the requirement of conforming to a standard widely supported across repository platforms that conveys implicit linking and relationship information amongst files in a common package.

Software developed under the name *MyTARDIS* facilitates data transfer from central (synchrotron) storage to local university instances and integration of private and public data dissemination. It provides a user-friendly interface encouraging deposition to TARDIS and its use as a data-publishing platform.

The early success of TARDIS is leading to extensions to other subject areas within the biosciences, an example of the OAIS principle mentioned in §[Sec sec2.1]2.1 that the ‘Designated Community’ is defined by the Archive, rather than the other way round.

**Figure 1:**
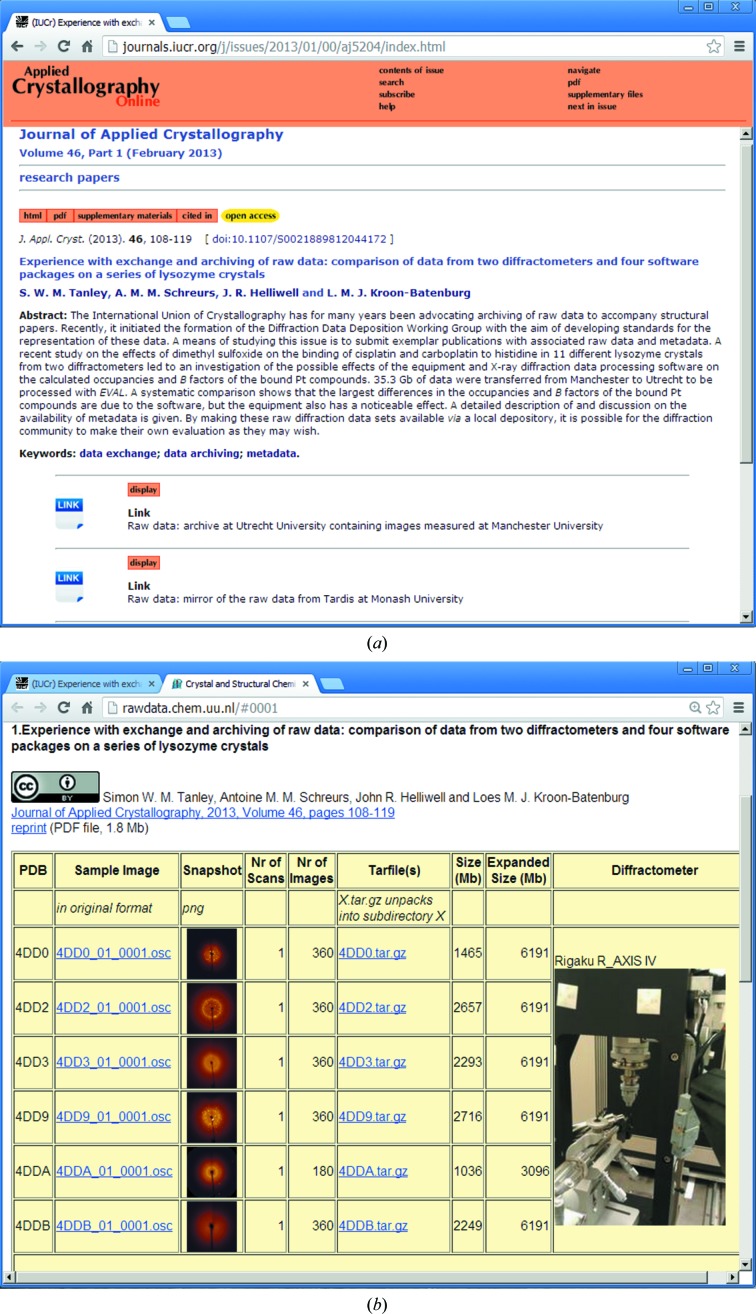

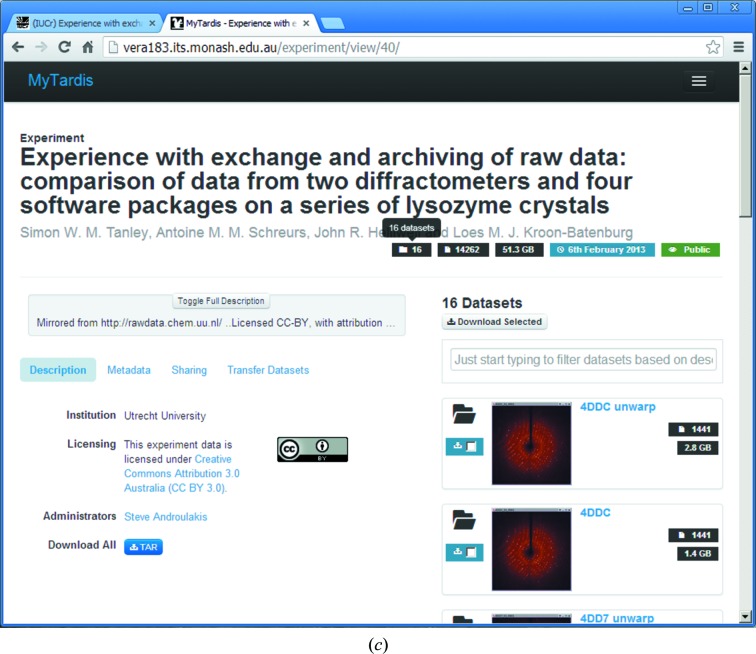
Example of a research publication (*a*) providing links (as supplementary materials) to copies of the experimental diffraction images (Tanley *et al.*, 2013 ▶) on a server at the University to which some of the authors are affiliated (*b*) and mirrored within the *MyTARDIS* system (*c*). The *MyTARDIS* interface summarizes the number of data sets, the number of files within each, the size of the individual data sets and access rights. It alo provides facilities to download individual data sets or the entire collection (but not individual files). On the local website established at their institution (*b*) the authors provide similar summary metadata, but have chosen also to supply photographs of the diffractometers used, which give additional information about the experimental geometry.

TARDIS has recently been used to mirror raw data sets hosted on a University server in Europe and referenced from a published article (Tanley *et al.*, 2013 ▶). Fig. 1 ▶ illustrates the links out from the publication to the experimental images hosted both at the University server and the *MyTARDIS* mirror.

A further exciting development based on *MyTARDIS* has been the establishment of the Australian Store.Synchrotron facility (https://store.synchrotron.org.au/). This cloud service provides instant, private online data access, sharing tools and self-service open data functionality to researchers. It was connected to the Australian Synchrotron’s X-ray beamlines in June 2013 and by late August 2014 was managing over 2.8 million raw diffraction images.

### 5.2. eCrystals   

eCrystals is an e-science platform developed by the University of Southampton for the National Crystallography Service (Coles *et al.*, 2006 ▶). Built on the *ePrints* institutional repos­itory software developed at Southampton (http://www.eprints.org), this could usefully act as a model for university data repositories that wish to make productive use of their deposited data sets. This project also has some first-hand experience in federation, with satellites established at some point in Sydney, Drexel and Birmingham. Their metadata development process (with JISC/UKOLN/IUCr participation) was a useful example of integrating a specific scientific discipline within a generic repository platform and has already been referenced in §[Sec sec4.2]4.2.

### 5.3. ReciprocalNet   

This is a mostly US federation of university crystallography departments collecting molecular structure information in a distributed database. It receives National Science Foundation funding as part of the National Science Digital Library project (http://www.reciprocalnet.org). At this stage in its development the focus is on sharing structures, visualization and other software tools and techniques, with a strong bias towards re-use for educational purposes. It has not systematically considered diffraction images. On the other hand, its establishment and maintenance has required investment in networking and synchronization technologies that will help to inform any approach to federated information management.

### 5.4. PDB   

The Protein Data Bank has already been discussed as an excellent exemplar of a well organized archive curated with considerable domain expertise. It has invested heavily in developing robust and highly respected practices in archiving and curating structural data. Many members of the protein crystallography community would regard it as a natural candidate for the centralized deposition of experimental data. However, as discussed in §[Sec sec3.2.1]3.2.1, it would require significant new funding to be able to perform this role, and considerable discussion of how far its remit should be extended if the community were to require a single central collection of raw data sets from all branches of crystallography.

### 5.5. Synchrotron and neutron facilities   

Most synchrotrons and other large experimental facilities have significant file-storage hardware and *ad hoc* data-retention policies. These are typically oriented towards the scientist, so that lost or corrupted data sets can be retrieved during the course of a scientific study. In many cases, facilities are retaining data for longer periods than publicly advertised, although in such cases it is common practice for data sets to be moved off disk platters onto magnetic tape for longer term storage.

The ISIS Neutron Source of the UK Science and Technology Facilities Council (STFC) provides an excellent example of a facility that implements a well designed and carefully managed data policy (http://www.isis.stfc.ac.uk/user-office/data-policy11204.html). This policy articulates the responsibilities of the facility, its staff and the different categories of users (commercial, academic, principal investigators, other researchers *etc.*). It stipulates data-retention times and policies and makes clear the current demarcation between responsibilities for archiving (*i.e.* secure storage and bit-level format migration) and content curation:

Section 4.2.3. These results will be stored long-term by the Facility. It will not be the responsibility of the Facility to fully curate this data *e.g.* to ensure that software to read / manipulate this data is available.

However, identification of data sets and their association with specific experiments (and subsequent publications) do form part of the Facility’s curation provision, using the Core Scientific Metadata Model developed within STFC (Matthews *et al.*, 2010 ▶).

It would be useful to recover such existing data sets for secure long-term storage before they are lost for good. However, this is really only ‘useful’ in cases where a sufficiently complete metadata package could be constructed, either from information already accompanying the stored data sets or recoverable from an experimental logs database.

### 5.6. High-throughput crystallography   

Recent initiatives (such as structural genomics) involving high-throughput crystallography have led to the construction of new beamlines and laboratories that have generated large volumes of raw data and associated structures. There has been a significant investment in new technologies, and an accompanying investment of effort in bioinformatics and data-handling procedures, in part to fulfil funding requirements for technology exchange.

The development of new laboratory-information systems such as ISPyB (Delagenière *et al.*, 2011 ▶) holds promise for the greater harmonization of workflow and data management between laboratories that use it. As with the operational framework of a facility such as ISIS (see §[Sec sec5.5]5.5), structured information models can only make it easier to associate raw data sets with the metadata required to characterize them fully and facilitate subsequent re-use.

We consider it unfortunate that the many structural genomics initiatives were launched without a consistent requirement for archiving across all participants. Nevertheless, individual laboratories or consortia have developed their own approaches to this. The Joint Center for Structural Genomics, for example, has established a repository of X-ray crystallo­graphic data sets, including full sets of diffraction images, for all of the structures that it has solved and deposited with the PDB. It specifically advertises that ‘these data sets are freely available to the scientific community for developing and testing new algorithms and benchmarking and teaching’ (http://www.jcsg.org/datasets-info.shtml). As of early 2014, the cited web page lists 19 references reporting research and development that has benefited from the use of this repository.

## 6. Conclusions   

This series of articles has been undertaken as part of the work of the Diffraction Data Deposition Working Group established at the 2011 IUCr Congress upon the initiative of former President Sine Larsen (http://www.iucr.org/resources/data/dddwg). Its members are John Helliwell (Chair), Tom Terwilliger, John Westbrook, Steve Androulakis, Sol Gruner, Loes Kroon-Batenburg, Hans-Josef Weyer and Brian McMahon, with consultants Alun Ashton, Herbert and Frances Bernstein, Gérard Bricogne and Bernhard Rupp.

From the case studies we see that current progress is relatively slow and uncertain. On the other hand, the initial implementations at eCrystals, ReciprocalNet and TARDIS have provided some experience with defining usable data packages. There has also been considerable progress in defining for diffraction images the most useful ‘characterization’ and ‘interpretation’ metadata (see §[Sec sec4]4). Undoubtedly more work will need to be performed to refine these specifications, depending on the actual technology platforms and communications infrastructure that the community chooses to implement. The existing efforts by wwPDB/EMDataBank (Validation) Task Forces and IUCr Commissions to specify ‘metadata for reproducibility’ have been very valuable, and should be built on further, especially where new and evolving techniques are involved.

For any resource to take on the centralized management of these data would require a clear community mandate and a significant long-term funding commitment.

Building a coherent collection strategy across the variety of possible repositories at present is challenging. Two steps would help at least to encourage and keep track of depositions in the near future.

(i) The community should issue a clear recommendation that DOIs be registered for deposited data sets and these DOIs be referenced in associated publications. Such a proposal was made by the Diffraction Data Deposition Working Group following its 2012 summer meeting in Bergen.
(ii) An effort should be made to create at least an informal list of registered DOIs for any data sets newly deposited. Initially, this could be performed by IUCr Journals for data sets referenced in their publications.

Leading on from the second, the community should work with DOI registration agencies (DataCite, CrossRef) to identify some mandatory metadata to be inserted in future data DOI registrations that would identify them as diffraction images for crystallographic structure determination. This could allow automated harvesting so that one could build a database of deposited crystallographic images.

IUCr Journals would be willing to contribute effort and expertise in such a project, but it would be preferable in the longer term that any central database of experimental data DOIs should be managed by some agency with wider interests rooted in the community. The IUCr Commissions have an important role to play in this.

There remains the wider challenge of raising the general level of community awareness of all aspects of systematic deposition and retention of experimental data. We hope that this article, and the others in this series, bring to the working crystallographer a greater appreciation of the benefits of following new procedures that emerge as best practice in data characterization, annotation and validation, whether, in the end, they are retained for a shorter or a longer period of time.

## Figures and Tables

**Table 1:** Typical sustained data rates for detectors used for macromolecular crystallography at the National Synchrotron Light Source and Diamond Light Source beamlines compared with the expected rates from the Eiger detector, expressed as multiples of the typical data rate for an inexpensive USB disk of ∼200 Mb s^−1^ From Bernstein *et al.* (2013 ▶).

Detector	Raw image size (MB)	Frame rate (Hz)	Compressed rate (Gb s^−1^)	USB disk data rate
ADSC Q315 (2 × 2 binned)	18	0.37	0.013	0.07
PILATUS 6M	24	10	0.48	2.40
PILATUS 6M-F (fast)	24	25	1.2	6
PILATUS3 6M	24	100	4.8	24
Eiger 16M	72	125	18	90
